# Social determinants of homelessness from childhood to adolescence: protocol for a living systematic review and meta-analysis

**DOI:** 10.1136/bmjopen-2024-095164

**Published:** 2025-06-04

**Authors:** Jessica A Heerde, Craig A Olsson, Susan M Sawyer

**Affiliations:** 1 Department of Paediatrics, The University of Melbourne, Parkville, Victoria, Australia; 2 Centre for Adolescent Health, The Royal Children’s Hospital Melbourne, Parkville, Victoria, Australia; 3 SEED Lifespan Strategic Research Centre, School of Psychology, Deakin University, Burwood, Victoria, Australia; 4 Murdoch Children’s Research Institute, Parkville, Victoria, Australia; 5 Department of Social Work, The University of Melbourne, Parkville, Victoria, Australia; 6 School of Population Health, Curtin University, Perth, Western Australia, Australia

**Keywords:** Adolescent, PUBLIC HEALTH, EPIDEMIOLOGIC STUDIES, Risk Factors

## Abstract

**Abstract:**

**Introduction:**

Homelessness across the early life course poses a grave and largely preventable challenge. Those who experience homelessness early are at increased risk of a range of health, education and social inequities that can extend across the life course. Better understanding of the modifiable factors on the pathway to homelessness is needed to inform prevention at the earliest possible point in the life course and reduce the rate of child and adolescent homelessness at the population level. Here we address this gap in real-time knowledge by establishing a living systematic review of population studies of social determinants of homelessness from childhood to adolescence.

**Methods and analysis:**

We will search MEDLINE, Embase, PubMed and PsycINFO for population-based prospective cohort studies reporting on social determinants of child and adolescent homelessness from 0 to 24 years. No study eligibility restrictions will be placed on the date, country of origin or language of publications. Study quality will be assessed using the Methodological Standards for Epidemiological Research scale. Associations between social determinants and homelessness will be reported using pooled relative risk ratios with 95% CI. If sufficient data are available, the influence of subgroup and methodological factors will be examined using meta-regression.

**Ethics and dissemination:**

The study will synthesise published studies that have previously been granted ethics approval, meaning that it is exempt from ethics review or approval. Study findings will be disseminated through a peer-reviewed journal article, conference and seminar presentations. The authors will distribute a plain language summary through their academic and professional networks.

**PROSPERO registration number:**

CRD42024577716.

**STRENGTHS AND LIMITATIONS OF THIS STUDY**

- This living systematic review protocol is reported in accordance with the Preferred Reporting Items for Systematic Reviews and Meta-analysis Protocols and is registered with PROSPERO (CRD42024577716).
- The review methodology has been specified a priori, and a comprehensive search strategy across four key health, medicine and biomedical databases has been developed in consultation with a research librarian.
- This review will bring together the global evidence on drivers of homelessness from childhood to adolescence (0–24 years, inclusive of high, middle, low income countries), based on quality data from population-based longitudinal studies (sampled at baseline to be representative of populations).
- Most studies are expected to be observational, and it is anticipated that the identified studies will have limited capacity to draw causal inferences.
- A small number of population-based prospective cohort studies may preclude our planned meta-analysis.

## Introduction

 Childhood and adolescence are critical periods of growth and development that set a foundation for healthy adult development. There has been much progress in improving the health of children and adolescents worldwide,^1 2^ yet current global circumstances, including rising living costs and inflation, natural disasters and climate-driven displacement, war and political conflict, and poverty and income inequality heighten the risk conditions that children and adolescents are born into and grow up in. Homelessness and inadequate housing further compound these risk conditions.^3^

The Institute of Global Homelessness^4^ estimates over 1.6 billion people are inadequately housed; rates of homelessness among children and adolescents continue to increase in many Organisation for Economic Co-operation and Development^5^ member countries. Homelessness places children and adolescents at markedly increased risk for early mortality and preventable multimorbidities.^6^,^8^ This presents a strong imperative for action to prevent child and adolescent homelessness by identifying the earliest opportunities in the life course to invest in effective, preventive interventions. To do this requires a robust foundation of scientific knowledge about the modifiable social and economic determinants of early life homelessness, that is those determinants that can be targeted or changed through intervention or prevention efforts.

We know of only two prior systematic reviews that have attempted to bring together the global literature on drivers of homelessness in childhood and adolescence. The first is a systematic review and meta-analysis, published in 2016, of causes of homelessness for 0–24 years between 1990 and 2013.^9^ Across the 49 identified peer-reviewed studies, the authors defined six frequently reported reasons for homelessness: poverty (eg, housing instability), family conflict (eg, domestic violence), abuse (eg, maltreatment), psychosocial health (eg, mental health) and delinquency (eg, conflict with the law), as well as a group of other reasons. The second is a more recent systematic review, published in 2022, that analysed data from 16 studies examining risk and resilience factors for homelessness in 0–25 years between 1950 and 2020.^10^ Across these peer-reviewed studies, difficulties with family, mental health or substance use problems, and a history of problem behaviours were identified as risk factors for homelessness, while a supportive family and high socioeconomic status were protective factors. Studies within the 2022 review were conducted predominately in the USA. While studies in that review were required to include a comparison group of non-homeless participants, they focused on adolescents (>11 years) and almost 40% used cross-sectional study designs. A meta-analysis was not conducted.^10^ In contrast, studies within the 2016 review were primarily conducted in low-middle-income countries (Africa, Asia, South America and Eurasia).^9^

Studies of adolescent homelessness included in these prior reviews were limited by difficulties in sampling, engaging participants and tracing samples. These were largely cross-sectional studies (quantitative and qualitative) and reported data on adolescents experiencing or at high risk for homelessness, without a comparison group who were not experiencing homelessness. The cross-sectional study design means that estimates for factors that contributed to homelessness over time or that were stratified by age or stage of child and adolescent development could not be calculated. Despite the breadth of data analysed in prior reviews, they could not robustly examine modifiable factors on the pathway to homelessness, because they lacked information on relevant exposures over key developmental periods.

Homelessness for children and adolescents is largely preventable. Traditionally, global, regional and national responses to tackling child and adolescent homelessness are reactive rather than systemic, meaning that they are overwhelmingly ‘downstream’ and are almost exclusively focused on individuals or families who are at imminent risk of homelessness.^11^,^13^ This approach requires significant investment of resources at federal, state and local levels. Prior reviews have drawn attention to a lack of rigorous evaluative evidence for many of the downstream programmes and responses relied on by communities and governments internationally to address youth homelessness, and research-implementation gaps around existing data^12 14^ and policy action on child and adolescent homelessness. Better understanding of the modifiable factors on the pathway to homelessness is needed to move ‘upstream’ to inform prevention at the earliest possible point in the life course and reduce the rate of child and adolescent homelessness at the population level.^15^ Opportunities to generate this knowledge are afforded through the analysis of data collected within longitudinal population-based cohort studies that span the developmental periods of childhood, adolescence and adulthood.^15^ For this reason, there is a strong case for synthesising this international literature and quantifying the association between social determinants in childhood and adolescence and homelessness in adulthood.

The purpose of this review is to address prior review limitations by establishing a living systematic review^16^ of the global evidence on pathways to homelessness (inclusive of high, middle, low income countries), based on quality data from population-based prospective cohort studies (sampled at baseline to be representative of populations), that enable detailed developmental mapping of risk and protective factors from childhood to adolescence (0–24 years). Our aims are threefold: (1) track modifiable social determinants that place children and adolescents at increased risk of homelessness, (2) track modifiable social determinants that place children and adolescents at reduced risk of homelessness and (3) sequence social determinants developmentally such that opportunities to intervene are clearly marked at each age and stage of development from childhood into adolescence (0–25 years). We will maintain this as a living review, updated annually, to ensure that the most recent evidence on early pathways to homelessness is captured and made available to applied practice and policy makers with a commitment to eliminating the drivers of homelessness in children and adolescents worldwide.

## Methods and analysis

### Protocol and registration

This protocol is registered with the International Prospective Register of Systematic Reviews (PROSPERO, CRD42024577716) and is reported in accordance with the Preferred Reporting Items for Systematic Reviews and Meta-analysis Protocols.^17^

### Participants

This review will include population-based prospective cohort studies examining the social determinants of child and adolescent homelessness (see Outcome measures section). This review will use the definitions of childhood and adolescence that, increasingly applied in framing social policies and service systems, conceptualise childhood and adolescence as spanning from 0 to 9 years and 10 to 24 years, respectively.^18^

### Search strategy

Searches will be performed in the following electronic health, medicine and biomedical, and social science databases of peer-reviewed research: MEDLINE, Embase, PubMed and PsycINFO. To ensure uniformity in search terms across each database, searches will use variations and combinations of database-specific controlled subject vocabulary (ie, MeSH terms and subject headings) relating to children and adolescents, social determinants and homelessness (refer to the sections on Exposure and Outcome measures). The use of MeSH terms allows for search terms to be examined as subject headings (eg, ill-housed persons) and expanded to retrieve records that include synonyms and narrower terms indexed to subject headings (eg, homeless youth, refugee camps, street youth, unhoused persons). Databases will be searched from their inception until the date of the search. No restrictions will be placed on the location of eligible studies. The search strategy for MEDLINE (Ovid) is presented in table 1. The search strategy was developed in consultation with a health librarian at the Royal Children’s Hospital in Melbourne, Australia.

**Table 1:** MEDLINE search strategy

Set	Search statement
1	exp *ill-housed persons/
2	(Homeless* or street-youth* or street-child* or street-sleep* or street-involve* or runaway* or unsheltered or no-fixed-abode or unhoused or temporary-housing or insecure-housing or emergency-housing or ill-housed or unstably-housed or shelterless or street-people or living-on-the-streets or vagabond* or vagrant*).tw,kf.
3	1 or 2
4	(newborn* or new-born* or baby or babies or neonat* or neo-nat* or infan* or toddler* or pre-schooler* or preschooler* or kinder or kinders or kindergarten* or kinder-aged or boy or boys or girl or girls or child* or pediatric* or paediatric* or juvenile* or minor or minors or underage* or under-age* or school-age* or schoolage* or schoolchild* or schoolgirl* or schoolboy* or pre-teen* or preteen* or tween or tweens or pre-adolescen* or preadolescen* or pre-puberty or prepuberty or pre-pubescen* or prepubescen* or adolescen* or youth or youths or teen or teens or teenage* or student* or young-adult* or young-people* or young-person* or emerging-adult* or emerging-people* or emerging-person* or AYA or AYAs or CAYA or CAYAs).tw,kf,hw.
5	*risk/ or exp *risk assessment/ or exp *risk factors/
6	*social vulnerability/
7	*protective factors/
8	(predict* or risk or risks or protective-factor*).tw,kf.
9	5 or 6 or 7 or 8
10	exp cohort studies/
11	(longterm or long-term or repeat* or serial or longitudinal* or follow-up or follow-up or cohort* or retrospective* or prospective*).tw,kf.
12	10 or 11
13	3 and 4 and 9 and 12
14	limit 13 to (case reports or comment or editorial or guideline or letter or practice guideline or preprint)
15	13 not 14

### Inclusion criteria

Eligible studies will be peer-reviewed population-based prospective cohort studies, as defined in the Cochrane study design guide.^19^ We will include studies where participants (a subsample of) have experienced homelessness and where exposure to modifiable social determinants preceded the homelessness outcome measured. For example, studies will compare the risk of homelessness following exposure to social determinants for participants who have experienced homelessness and participants who have not experienced homelessness. We will require exposure to social determinants to have occurred at least 6 months prior to experiencing homelessness for the study to be included. Studies will report at least one estimate of the contribution of modifiable social determinants to increased or decreased homelessness or provide sufficient information to allow the calculation of an estimate. Social determinants that will be considered for inclusion are described below (refer to the section on Outcome measures). Study eligibility will be restricted by the age of the sample at baseline: study participants will be limited to 0–24 years. No restrictions will be placed on the date of the study, its country of origin or the language of publications. Abstracts of non-English language publications will be translated into English and reviewed for eligibility.

### Exclusion criteria

Studies reporting on social determinants of participants whose residence is (or has been) threatened due to financial insecurity or poor living standards, and who do not report homelessness (as described below), will be excluded. We will not include studies that analyse only concurrent exposure to social determinants and homelessness (ie, cross-sectional studies). Qualitative studies, case reports and case series, case-control studies, commentaries, editorials or opinion pieces, study protocols, practice guidelines and letters will be excluded. Clinical cohorts will be excluded to ensure the review findings are generalisable to the general population. Grey literature (book chapters, non-peer reviewed studies and government reports/guidelines), as well as dissertations and conference abstracts, will also be excluded. Prior literature reviews will be excluded; however, the reference lists of all excluded reviews will be screened to identify any additional relevant studies not identified through our search strategy.

### Outcome measure

The primary outcome being examined is homelessness. Included studies will report on homelessness as an outcome to be eligible for inclusion. We acknowledge there is no globally accepted definition of child and adolescent homelessness, with different typologies of homelessness used within high, upper middle and low income countries.^20 21^ For this review, homelessness includes children and adolescents who report a history of: (a) being unsheltered (eg, living directly on the streets or in spaces not intended for habitation); and/or (b) residing in emergency shelter (eg, crisis housing); and/or (c) residing in temporary accommodation (eg, short-term accommodation such as a rooming house, under the care/supervision of institutions); and/or (d) living in insecure and/or inadequate housing (eg, couch surﬁng).^21 22^ There will be no restrictions on the time frame in which homelessness is examined. Homelessness may be measured by self-report or administrative data (eg, homelessness service system records).

### Exposure measures

We will include population-based cohort studies that analyse exposure to social determinants and risk for homelessness in childhood and adolescence. Exposure measures will be examined across all systems defined by the Bioecological Model of Human Development.^23 24^ This includes exposures at the level of the child and adolescent as well as the broader social ecologies within which children and adolescents live and grow, with a focus on family, peer, school, digital and community-level microsystems, connections between microsystems (meso-system), broader societal workplace, health, education and employment institutions (exo-system) and master narratives of society related to gender, race and socioeconomic position (macro-system). Exposures may be measured by self-report, familial-report or administrative data (eg, hospital and emergency departments, education, youth justice system or social security records).

### Study selection

We will import the full publication details and abstracts for all studies identified through the search of electronic databases into EndNote reference management software,^25^ where duplicates will be removed. The remaining citations will then be imported into Covidence systematic review management software for screening.^26^ To ensure that the inclusion and exclusion criteria are consistently applied and in line with best practice guidelines,^27^ the title and abstract of all identified studies will be independently screened by two members of the research team. Where there is uncertainty related to study inclusion between the two team members, a third member of the research team will examine the study title and abstract. Cohen’s kappa statistic will be used to calculate the inter-rater reliability for the full title and abstract screening process.^28^ Where reliability is low (<0.40), the research team will review and revise the eligibility criteria and double-code a random 10% of retrieved studies before recalculating Cohen’s kappa statistic. If applicable, revised eligibility criteria will then be used to guide the revised title and abstract screening process. Next, full text screening of all remaining studies will be independently performed by two members of the research team in line with the eligibility criteria. A third team member will resolve any uncertainty related to study inclusion between the two team members. Where clarification is needed to determine if a study is eligible for inclusion, the original study author(s) will be contacted twice, via email. The study will be excluded if no response is received from the authors. If there are multiple (two or more) eligible studies that report analyses from the same dataset, the study with the longest follow-up duration will be included.

### Data extraction

Data will be extracted from all studies identified for inclusion in the review. Data extraction will be independently performed by at least two members of the research team using a standardised, prespecified form (Excel) developed by the research team. A third team member will resolve any uncertainty related to data extraction and also check the data extracted to identify and correct any errors. Where important data on the exposures or outcome is incomplete or missing, the original study author(s) will be contacted twice, via email. The study will be excluded where there is no response received from the authors. A summary of potential data extraction fields is presented in table 2.

**Table 2:** Summary of potential fields for inclusion in the data-extraction form

Study identification information	Outcome/exposure conditions
Study details (eg, author(s), year of publication, journal name)	Type of homelessness (ie, unsheltered, residing in emergency shelter)
Study country of origin	Age at which homelessness is measured
Cohort study name	Sample size (homeless participants)
Study design	Sample characteristics (homeless participants, for example, age, sex, race, ethnicity)
Year(s) of study	Predictors of homelessness with measures of association (eg, RRR, OR, HR, 95% CIs)
Study setting	Time period between exposure and outcome
Sample size	Significance levels (p values)
Sample characteristics (eg, age, sex, race, ethnicity, other demographic characteristics of interest)	Subgroup analysis results
Year data collection commenced/ended (eg, length of follow-up)	Measures of bias/sensitivity analyses
Age at follow-up	
Source(s) of data (eg, self-report, familial-report, administrative data)	
Statistical methods used	
Attrition rates/Loss to follow-up	
Funding sources declared	
Conflicts of interest declared	

RRR, relative risk ratio.

### Risk of bias assessment

Study quality and bias will be assessed using the Methodological Standard for Epidemiological Research (MASTER) scale^29^ and independently performed by two members of the research team. A third team member will resolve any conflicts in the evaluation of bias. Studies assessed as being of low quality will be retained in the analysis and a sensitivity analysis performed restricting the meta-analysis to those studies which are above the median MASTER score^29^ (refer to the section on Data synthesis and statistical analysis).

### Data synthesis and statistical analysis

We will provide a detailed descriptive synthesis of all studies included in the review, including study identification information, types of measures used to assess both exposure to social determinants and child and adolescent homelessness, measures of association and the quality of the analysed studies.

The analysis strategy will directly map to review aims and will be developed by the author team and in consultation with a biostatistician within the Clinical Epidemiology and Biostatistics Unit at the Murdoch Children’s Research Institute, Melbourne, Australia. Where there are sufficient data within the reviewed studies and methodological/conceptual heterogeneity is not excessive, a meta-analysis will be conducted to produce pooled estimates. Associations between social determinants and homelessness will be reported using pooled relative risk ratios with 95% CI. The *I*² statistic will be used to examine heterogeneity between studies; a value greater than 50% will be considered to indicate substantial heterogeneity.^30^ A funnel plot and Egger’s test will be used to assess potential publication bias.^31^ Where there is sufficient data in the reviewed studies, the influence of subgroups and methodological factors on social determinants or homelessness will be examined using meta-regression. Subgroups may include: sex, age, study country of origin and country type (eg, high income), and type of homelessness (eg, unsheltered, residing in emergency shelter). Sensitivity analyses will be conducted to examine the effect of study quality on the study outcomes; studies assessed as being of low quality (defined as a quality assessment score below the median on the MASTER scale) will be excluded.^29^ The software used to conduct the analysis will be STATA. If there are insufficient data for a meta-analysis to be performed, the quantitative evidence presented in the reviewed studies will be summarised using a narrative approach.^32^

### Patient and public involvement

There will be no patient or public involvement in the design or conduct of this review. There is lived experience of homelessness within the author team. Project findings will be reported and disseminated in consultation with peak homelessness bodies and social service and policy leaders that advocate for, and provide support to children, adolescents and families who are at risk of or who experience homelessness.

### Ethics and dissemination

The systematic review and meta-analysis will be conducted on published studies that have already obtained approval from their human research ethics committee or institutional review board, meaning that it is exempt from ethics review or approval.

Despite recognition that effective prevention programming to address health and social outcomes is highly cost effective,^33 34^ there have been few attempts to systematically review what is known about social determinants of homelessness from childhood to adolescence, which continues to significantly limit progress in implementing best practice, evidence-based, approaches to reducing the rate of homelessness at the population level. This study protocol and the findings of the review will be published in an appropriate international peer-reviewed journal. Review findings will be presented at national and international conferences/symposia and through seminar presentations. In establishing this review as a ‘living systematic review’, we will develop a parallel ‘living research brief’ that links to the published review. Our intention is to re-run the search and screen for new literature on an annual basis. We will attempt to maintain this as a living review without end date. We will disseminate our findings through our professional networks to stakeholders in the systems working with children and adolescents, including relevant professional organisations and policy makers in Australia and internationally and social service and policy leaders that advocate for homelessness prevention. Both the published protocol and review will be placed within our University institutional repository. Links to the published review and living research brief will be circulated via social media.
