# Supplementary material for: Social determinants of homelessness from childhood to adolescence: protocol for a living systematic review and meta-analysis
Source: BMJ Open. 2025 Jun 4;15(6):e095164. doi: 10.1136/bmjopen-2024-095164 (PMC12142096; doi:10.1136/bmjopen-2024-095164)
Supplement: Supplementary file 1 [file bmjopen-2024-095164.reviewer_comments.pdf]

PEER REVIEW HISTORY

BMJ Open publishes all reviews undertaken for accepted manuscripts. Reviewers are asked to complete a checklist review form and are provided with free text boxes to elaborate on their assessment. These free text comments are reproduced below.

ARTICLE DETAILS

Title (Provisional)

Protocol for a living systematic review and meta-analysis of social determinants of homelessness from childhood to adolescence.

Authors

Heerde, Jessica; Olsson, Craig A; Sawyer, Susan M

VERSION 1 - REVIEW

**Reviewer**	**1**
**Name**	**Williamson, Andrea**
**Affiliation**	**University of Glasgow, GPPC, School of Medicine, Dentistry and Nursing, MVLS**
**Date**	**11-Feb-2025**
**COI**	**None**

This is an important topic which as is described has been grappled with for a long time. There is however a premise in the paper which I think is inaccurate which is that if we only know more from the research evidence then this will change the approach to tackling children and young people becoming homeless. In my experience we do know a lot- the issue is a massive implementation gap across countries and settings, largely driven by the social determinants of health. So I don't think this claim can be made unless you can clearly set out what this particular piece of work is going to acheive which others have not to date.

The aim to consider the modifiable determinants across all country context settings is really positive. However the search terms then need to be carefully considered to include children and young people who are living in refugee camps- which drives a significant chunk of the global estimates.

I am also concerned by the narrow focus on prospective studies which means this will then exclude much of the global South and means you will not manage to meet your study aim. I would need to see much more justification of why this approach is being taken, also with some scoping of the existing studies across the globe to persuade the reader this is achievable.

I would also need to see justification as to why the work is not considering the whole range of research methods papers including qualitative ones, given the ambition set out in the paper.

There also needs to be set out clearly what you mean precisely by 'modfiable' or 'malleable' social and economic determinants to help the reader understand the 'so what' of the project (which is linked to the first concern about the implementation gap).

**Reviewer**	**2**
**Name**	**Gutwinski, Stefan**
**Affiliation**	**Charite Universitatsmedizin Berlin**
**Date**	**17-Mar-2025**
**COI**	**None**

Well designed protocoll. A short remark: Most studies will report data of participants of <24 and >24 age. It will be nealy impossible to avoid that. This should be described in the final publication.

VERSION 1 - AUTHOR RESPONSE

Reviewer 1

1. This is an important topic which as is described has been grappled with for a long time. There is however a premise in the paper which I think is inaccurate which is that if we only know more from the research evidence then this will change the approach to tackling children and young people becoming homeless. In my experience we do know a lot- the issue is a massive implementation gap across countries and settings, largely driven by the social determinants of health. So I don't think this claim can be made unless you can clearly set out what this particular piece of work is going to acheive which others have not to date.
   Thank you for these observations and we can but only agree with your reflections on the implementation gap. Homelessness across the formative years of childhood and adolescence is a topic that communities continue to grapple with, influenced by a range of social and economic determinants. As we note in our Introduction, current responses to tackling homelessness are largely drawn on research evidence derived from cross-sectional studies analysing data from adolescents experiencing or at high risk for homelessness, without a comparison group who were not experiencing homelessness. To clarify, this living review will be the first to systematically review population-level studies of upstream risk and protective factors for homelessness across childhood and adolescence. This piece of work will synthesise the existing evidence to inform prevention efforts. Notwithstanding the implementation gap around existing data, there is still concern that lack of synthesis of these upstream factors has blocked progress on upstream prevention efforts.

2. The aim to consider the modifiable determinants across all country context settings is really positive. However the search terms then need to be carefully considered to include children and young people who are living in refugee camps- which drives a significant chunk of the global estimates.
   We agree that there is significant variation in the way that homelessness is conceptualised internationally. To capture this variation, our search will use MeSH terms and subject headings. The use of MeSH terms and subject headings means that the various synonyms of homelessness/different terms used to describe homelessness in the literature and in different country contexts are automatically included in the search query. This is permitted through the inclusion of lines 1 and 2 in our search strategy (refer to Table 1). Terms used for 'homelessness' are categorised under the MeSH term 'ill-housed persons'. Retrieval of studies reporting various terms for homelessness is permitted through the inclusion of line 2 in our search strategy ('tw,kf' in Table 1). The inclusion of this line permits a search for author supplied keywords irrespective of the MeSH term assigned to a study. This line permits the retrieval of studies reporting on various forms of homelessness (of which, for example, refugee camps are included).

3. I am also concerned by the narrow focus on prospective studies which means this will then exclude much of the global South and means you will not manage to meet your study aim. I would need to see much more justification of why this approach is being taken, also with some scoping of the existing studies across the globe to persuade the reader this is achievable.
   Thank you for raising these queries. As noted throughout our manuscript, to progress developmentally informed prevention approaches, acknowledging that risk for homelessness is likely to be shaped by experiences from birth and across childhood and adolescence, and within the multiple systems in which children and adolescents interact, a focus on prospective studies is needed. The authors are aware of >10 published studies likely to meet our inclusion criteria. The search strategy will be designed to capture prospective studies published in a global context. While this approach to rigour is far more likely to identify papers in the global North, including the global South, we believe that this review will provide a rare opportunity to scope where existing prospective studies have (or have not) been conducted across the globe, which of itself will be an important finding. As mentioned above, a living review is as valuable for identifying what we don't know (i.e., where gaps in data and knowledge exist), as it is for centralising what we do know. In identifying gaps, such as a potential lack of prospective data from the Global South, our review findings will be well positioned to help advocate for new data collections in areas of unmet need.

4. I would also need to see justification as to why the work is not considering the whole range of research methods papers including qualitative ones, given the ambition set out in the paper.
   To clarify, our aim is synthesise the evidence on early pathways to homelessness by mapping modifiable social determinants that increase/decrease risk of homelessness developmentally from childhood into adolescence. This aim can be most effectively achieved by synthesising data from longitudinal studies that enable temporal separation of exposures and outcomes. Longitudinal studies further minimise retrospective recall bias through prospective data collection as well as confounder bias through rich contextual data collections. These considerations and more make prospective data the preferred data resource from which to base new policy designed to prevent homelessness across the formative years of life.

5. There also needs to be set out clearly what you mean precisely by 'modfiable' or 'malleable' social and economic determinants to help the reader understand the 'so what' of the project (which is linked to the first concern about the implementation gap).
Thank you for this suggestion. To clarify, we define modifiable determinants as those that can be changed through intervention or prevention efforts to reduce risk of homelessness (for example, access to education or health care). We have clarified this on page 5. For consistency, we have replaced the term malleable with modifiable throughout the manuscript.

Reviewer 2

1. Well designed protocol. A short remark: Most studies will report data of participants of <24 and >24 age. It will be nearly impossible to avoid that. This should be described in the final publication.
We thank the reviewer for their comments. Thank you for raising this topic. As our focus is on social determinants in childhood and adolescence, study eligibility will be limited to those with participants 0-24-year-old at baseline (see page 9, line 8).

VERSION 2 - REVIEW

**Reviewer**	**1**
**Name**	**Williamson, Andrea**
**Affiliation**	**University of Glasgow, GPPC, School of Medicine, Dentistry and Nursing, MVLS**
**Date**	**02-May-2025**
**COI**	

Thanks for your responses to the review and I appreciate the changes you have made. It is clear from your responses that the team understands the nuances of the context that this work is to be conducted in. However the paper as it stands as I assess it still puts far too much emphasis on this work being a way to solve homelessness. Some sentences acknowledging the following in the paper are required. 1. That quantitative data and especially prospective studies- which are really hard to do - that's why they are not often done- are only one level of evidence in this field. There are others but you then can explain why you have focussed and are going to use the methods you are using- which are clear and justified. 2. Acknowledge the implementation gap between research and practice- as it stands the paper comes across as going to solve everything, which of course (regrettably) it is not going to.

3. Please include mention of refugee camps in the text not just buried in the mesh terms. As its an important global context to make reference to to be accurate.

VERSION 2 - AUTHOR RESPONSE

Reviewer 1

1. Thanks for your responses to the review and I appreciate the changes you have made. It is clear from your responses that the team understands the nuances of the context that this work is to be conducted in. However the paper as it stands as I assess it still puts far too much emphasis on this work being a way to solve homelessness. Some sentences acknowledging the following in the paper are required.
   We thank the Reviewer for their feedback and suggestions on our manuscript. We have further deemphasised the possibility that this review will solve homelessness; it was never our expectation that a single systematic review could do this. Rather, our ambition is to create a living repository of the global evidence on risk and place this information in the hands of policy makers and practitioners working to end homelessness.

2. That quantitative data and especially prospective studies- which are really hard to do - that's why they are not often done- are only one level of evidence in this field. There are others but you then can explain why you have focussed and are going to use the methods you are using- which are clear and justified.
   We agree that prospective studies of homelessness are rare because they are so hard to undertake. This is why we have expanded the scope of our search to findings from any population-based cohort study that has measured homelessness and upstream risk and protective factors across childhood and adolescence. We do recognise that other sources of data (e.g., cross sectional and qualitative) are also valuable; however, the temporal nature of cohort data uniquely positions them to map pathways to homelessness, and determinants thereof, in ways that can powerfully inform public health and health services approaches to reducing homelessness. We have noted in our Introduction the evidence provided by prior studies (see page 6, paragraph 2).

3. Acknowledge the implementation gap between research and practice- as it stands the paper comes across as going to solve everything, which of course (regrettably) it is not going to.

   We have included additional text noting the implementation gap around existing data (see page 7, paragraph 1).

4. Please include mention of refugee camps in the text not just buried in the mesh terms. As its an important global context to make reference to be accurate.
   In response to this suggestion, we have updated the text in the 'Search strategy' section to mention refugee camps.
